# Measuring Food Brand Awareness in Australian Children: Development and Validation of a New Instrument

**DOI:** 10.1371/journal.pone.0133972

**Published:** 2015-07-29

**Authors:** Laura Turner, Bridget Kelly, Emma Boyland, Adrian E. Bauman

**Affiliations:** 1 School of Health and Society, Faculty of Social Sciences, University of Wollongong, Wollongong, NSW, Australia; 2 Early Start Research Institute, Faculty of Social Sciences, University of Wollongong, Wollongong, NSW, Australia; 3 Department of Psychological Sciences, Institute of Psychology, Health and Society, University of Liverpool, Liverpool, United Kingdom; 4 Prevention Research Collaboration, Sydney School of Public Health, University of Sydney, Sydney, NSW, Australia; TNO, NETHERLANDS

## Abstract

**Background:**

Children’s exposure to food marketing is one environmental determinant of childhood obesity. Measuring the extent to which children are aware of food brands may be one way to estimate relative prior exposures to food marketing. This study aimed to develop and validate an Australian Brand Awareness Instrument (ABAI) to estimate children’s food brand awareness.

**Methods:**

The ABAI incorporated 30 flashcards depicting food/drink logos and their corresponding products. An abbreviated version was also created using 12 flashcards (ABAI-a). The ABAI was presented to 60 primary school aged children (7-11yrs) attending two Australian after-school centres. A week later, the full-version was repeated on approximately half the sample (n=27) and the abbreviated-version was presented to the remaining half (n=30). The test-retest reliability of the ABAI was analysed using Intra-class correlation coefficients. The concordance of the ABAI-a and full-version was assessed using Bland-Altman plots. The ‘nomological’ validity of the full tool was investigated by comparing children’s brand awareness with food marketing-related variables (e.g. television habits, intake of heavily promoted foods).

**Results:**

Brand awareness increased with age (p<0.01) but was not significantly correlated with other variables. Bland-Altman analyses showed good agreement between the ABAI and ABAI-a. Reliability analyses revealed excellent agreement between the two administrations of the full-ABAI.

**Conclusions:**

The ABAI was able to differentiate children’s varying levels of brand awareness. It was shown to be a valid and reliable tool and may allow quantification of brand awareness as a proxy measure for children’s prior food marketing exposure.

## Introduction

Childhood obesity is a major public health issue in Australia and worldwide [1]. One in four Australian children (2–17 years) are overweight or obese, based on measured data [2]. In one decade, prevalence of overweight and obesity in Australian children nearly doubled: from 12% in 1985 to 20% in 1995 [1]. As these rapid increases in obesity prevalence cannot be attributed to genetic changes, research has progressively focused on the influence that environmental factors may have on weight gain [3]. Mass media has received increasing attention for its influence over the socio-cultural aspects of the food environment and a major component of this is the infiltration of pervasive food marketing into daily living [3, 4].

Increased prevalence of childhood obesity has paralleled rising levels of food marketing, typically dominated by unhealthy food products [5]. Research in several countries has identified that the product categories most commonly promoted to children include: confectionary, sweetened cereals, fast food, salted/savoury snacks and soft drinks (known as the ‘big 5’) [4]. These promotions undermine government nutrition guidelines and provide conflicting messages regarding nutrition to children [5]. Consequently, restricting children’s exposure to unhealthy food marketing is recognised as a global health priority, including by the United Nations [6] and the World Health Organization [5]. Despite this, limited regulatory restrictions exist in most countries [7].

Most child-directed food marketing campaigns take a ‘branding’ approach and the concept of ‘brand awareness’ is identified in health, marketing and psychology literature for its potential impact on children’s food preferences and consumption behaviours [8–10]. The term ‘branding’ refers to a unique name or symbol that identifies the product of a seller and distinguishes it from that of competitors [8]. Food represents one of the most highly branded commodities, relative to other consumer items [11]. It is the intent of marketers to build ‘brand equity’, defined as a ‘set of assets linked to a brand name that add value to a brand’ [8]. Brand awareness is considered to be one of the most valuable assets linked to a brand and represents a core component of brand equity [8].

Brand awareness signifies an individual’s recognition that a brand belongs to a specific product category as well as their ability to recall brand names [8]. Brand awareness in childhood may form the basis for product attitudes and preferences later in life [12], and is a necessary circumstance for brand likeability and purchase requests to occur [13]. Empirical evidence also indicates that marketing exposure leads to changed preferences for, and choice of, related food brand as well as related food categories [14]. This is important from a population nutrition perspective, whereby exposures to unhealthy food marketing can lead to population shifts towards unhealthy dietary patterns, rather than simply changes between brands within an unhealthy food category. Brand awareness, either as explicit awareness or in implicit memory, has been proposed as an initial and important response to marketing exposures, from which other attitudinal and behavioural responses (e.g. food consumption) develop [1]).

Two mechanisms through which brand awareness is thought to impact on downstream responses to marketing exposure are that: (1) awareness provides an anchor to which other associations, such as positive product attributes and themes, can be attached; and (2) brand familiarity leads to liking and positive (affective) associations with the products [8].

The importance of brand familiarity on children’s food preferences was clearly demonstrated in an experimental trial by Robinson et al in 2007. In this study, children (*n* = 63, 3–5 years) were offered two paired samples of five identical foods/beverages, one at a time and asked to indicate which they preferred. One sample was wrapped in McDonalds packaging whilst the other was in plain packaging [15]. Children significantly preferred the taste of foods and drinks if they thought they were from McDonalds, and this effect was even evident for carrots (*p*<0.001). The importance of brands as anchors for other associations is highlighted through the child-targeted themes in food promotions aimed at children, which commonly include aspects of humour, action-adventure, fantasy and fun [16, 17].

Brand awareness may serve as a proxy measure of prior food marketing exposures, and may be predictive of future food choices and dietary habits [12]. However, few instruments exist to quantify cumulative brand awareness in children. The International Brand Awareness Instrument (IBAI) was first proposed in the US by Forman et al [18]. The tool consisted of a flashcard quiz (comprising 30 brands) that contained a recall and recognition task. The recall task exposed children to a brand logo and asked them to identify the brand name. The recognition task displayed pictures of differing food/drink products and asked children to choose the corresponding product [18]. The IBAI has since been adapted in Italy [19], Chile [20] and India [21]; these studies used a shorter version of the tool (12-brands) to reduce participant burden [19, 20, 21]. However, no thorough psychometric assessment of these tools has been conducted. While Franchin et al [19] assessed the agreement between the abbreviated tool and the full tool, this study did not consider the validity of the full version independently. Further, while many heavily promoted food brands are available internationally, variations in food brands exist across countries so geographically-specific versions of the tool can be adapted to suit different contexts.

This study aimed to develop an instrument adapted from the IBAI, referred to as the Australian Brand Awareness Instrument (ABAI), and to assess its validity and reliability amongst Australian children aged 7–12 years. A secondary aim was to create a shorter version of the tool and determine its validity relative to the longer version. If appropriate, this will provide a tool with decreased participant burden for use in later, more representative population samples, to quantify brand awareness. The ABAI seeks to assess relative and cumulative food/drink brand awareness in children rather than assess children’s awareness of any one particular brand. Relative brand awareness may be an indicator of children’s food marketing exposures [22] and may predict behavioural responses to food marketing. This information serves to strengthen evidence on the association between food marketing exposures and behavioural responses, via a cascade of knowledge, affect and behaviour [10] and can contribute to policy discussions to limit children’s exposure to unhealthy food marketing.

## Methods

### Selection of brands

With reference to previous IBAI studies [18–21], we developed the ABAI paying attention to specific criteria when selecting the brands. Firstly, we identified food/drink brands that represented the ‘big 5’ categories of foods and beverages. The final version contained equal numbers from each of these categories. These brands had to be accessible in supermarkets in the Illawarra region. A candidate pool containing 50 brands was generated, which was subsequently sent to four dietitians with expertise in food marketing research, in order to verify the ‘content’ validity of the instrument. The list was reduced to 30 brands based on this feedback (see S1 Table).

### Instrument development

**Fig 1:**
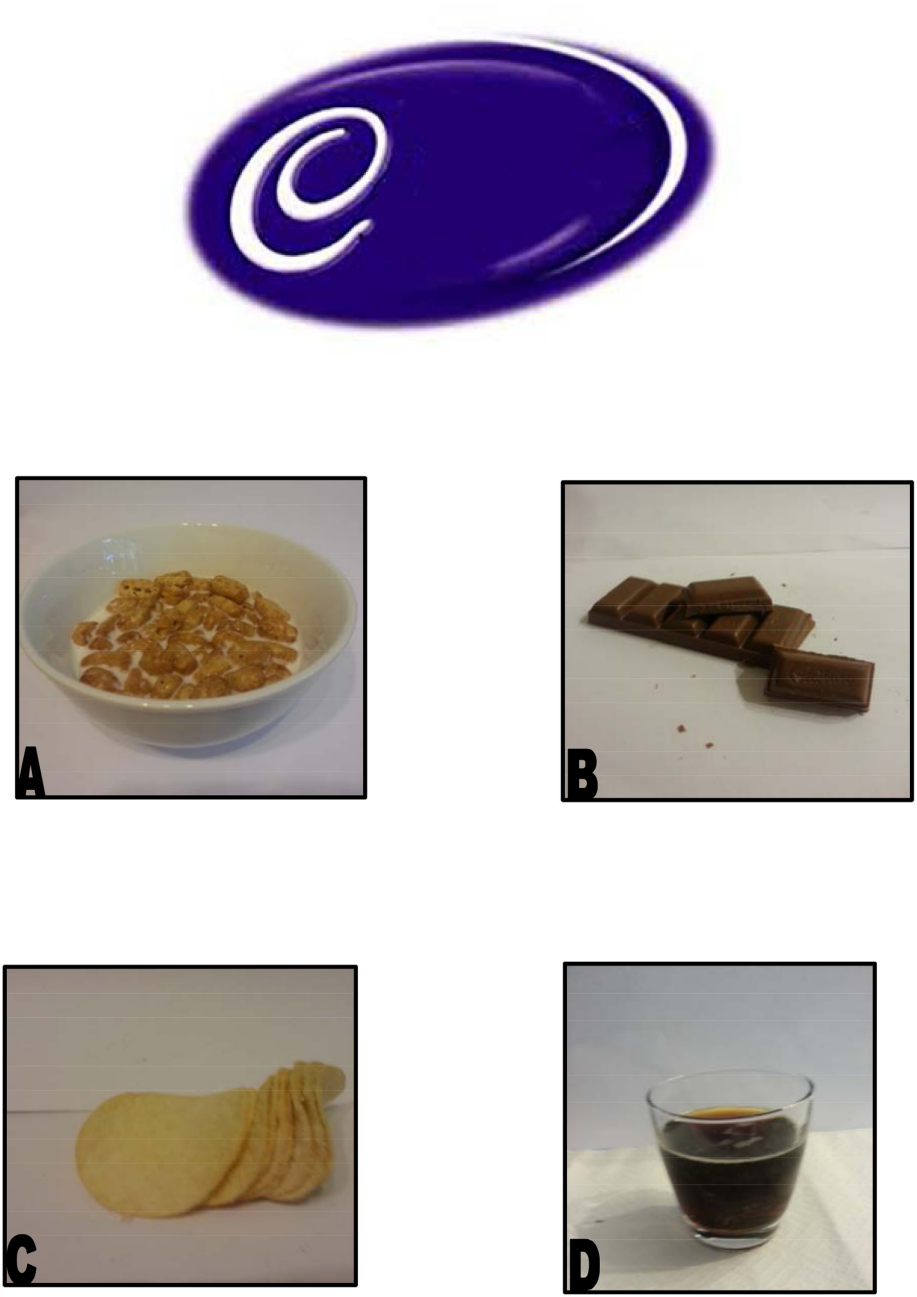
Sample flash card.

An age-appropriate instrument was developed using brand logos downloaded from company websites. These were de-identified by partially removing text from the logos using Adobe Photoshop CC v.12 software for Windows. Approximately 30% of included brands used a branded character instead of a logo; for example, Ronald McDonald was used instead of the golden arches symbol. The final tool used photographs of food and drinks, captured by the lead researcher (LT), to improve the authenticity of the recognition task. Four photographs were assigned to each brand (only one correct) for the recognition task. Researchers paid attention to the types of products contained in the recognition task and photos depicted only those foods that belonged to one of the ‘big 5’ products. In previous IBAI studies the selection of these products appeared arbitrary and not all could be categorised as a big 5 product. A sample flashcard can be found in Fig 1.

The recall task was contained on one side of the flash card, and the recognition task on the other. All 30 flash cards (containing 30 brand images and 120 food/drink pictures) were printed and laminated on A4 paper, and remained as separate cards to allow them to be shown in varied order. Brands for the abbreviated ABAI (ABAI-a) were selected based on the response distribution of the first 23 children to complete the full ABAI during the first interview, in order to represent a similar distribution.

### Questionnaire

A child questionnaire was designed to measure variables considered to be related to the measure of interest (brand awareness) and responses were used to assess the ‘nomological’ validity of the tool. The survey was developed by the investigators based on questions derived from the Schools Physical Activity and Nutrition Survey 2010 (SPANS) [23], and aimed to measure: i) basic demographic information (age, sex, postcode); ii) frequency of consumption of the ‘big 5’ foods and beverages (0 = never or rarely, 1 = 1–2 times a week, 2 = 3–4 times a week, 3 = 5–6 times a week, 4 = 1 time/day and 5 = 2 or more times/day); iii) amount of weekly TV viewing time (cumulative minutes/week), divided into commercial, non-commercial and pay TV, as well as additional TV-related variables, including the number of TVs in their household and frequency of watching TV whilst eating dinner; iv) frequency of supermarket attendance; and v) amount of weekly internet usage outside of school (cumulative minutes/week). The SPANS questionnaire was originally designed for self-completion by children aged from 11–12 years.

A parental questionnaire was developed using similar questions to the child questionnaire. We aimed for a parent-child paired sample, as the quality of self-report data is generally poorer for children younger than 14 years [24].

### Sampling

Children were recruited from two after-school childcare centres in the Illawarra region of New South Wales, Australia. These two childcare centres represented two areas with varying levels of Socio-Economic Status (SES) (one medium/high and one low). A sample of 80 children aged 7–12 years was initially selected. This age group has been shown to understand the ‘selling’ intent of advertising. However, not all are able to understand its ‘persuasive’ intent and this age group attach more importance to branding when it comes to food selection [25, 26]. The sample size was based on a previous IBAI study [20] and allowed the sample to be split into two groups for the assessment of reliability and agreement between the ABAI and ABAI-a.

### Procedures

The full-ABAI was administered individually to children along with the questionnaire at Interview 1. Children for whom written parental consent was obtained, and who provided verbal consent at the time of the interview, were led away individually for interview purposes. Firstly, a practice card was presented to the children containing a non-food brand. Once the children understood testing procedures, they were told the rest of the cards contained food/drink brands. For the recall task, children were asked to identify the name of the brand. For the recognition task, children selected the picture they believed matched the brand. Children were marked based on their responses. The children were then required to complete the child questionnaire, which was read out by the researchers (LT and BK). This process took approximately 10 minutes.

Each child completed two interviews each, at least one week apart. The sample was divided in half and children were alternatively assigned to repeat the full ABAI (‘reliability study’) or the ABAI-a (to assess agreement between ABAI and ABAI-a). Interview 2 followed the same procedures as the first (excluding the child questionnaire) and took approximately 5 minutes to complete.

Ethical approval for the study was obtained from the University of Wollongong Human Research Ethics Committee. Written parent informed consent was required for participation, as well as child verbal consent (obtained from BK and/or LT) at the time of data collection. Approval number: HE14/136.

### Statistical analyses

Analyses were conducted using IBM SPSS Statistical Software package version 21.0 for Windows (Armonk, NY: IBM Corp). The independent variables included: age, frequency of consumption of the ‘big 5’, amount of TV viewing (minutes/week) and other TV-related variables, frequency of supermarket attendance, and amount of internet usage (minutes/week). ‘Total screen time exposure’ was computed by combining the cumulative minutes/week of pay and commercial TV viewing and internet usage.

The frequency categories used for all ordinal variables in the questionnaire (frequency of consumption, watching TV while eating dinner, supermarket attendance) were collapsed into four broader categories (0 = Never or rarely, 1 = At least weekly (1–4 times/week), 2 = Almost daily (5–6 times/week), 4 = At least daily). An additional eating behaviour variable was created (‘total consumption score’) by adding together responses from this 4-item scale for the five food product categories. Using deciles of relative socio-economic disadvantage derived from Australian Bureau of Statistics (ABS) Socio-economic Indexes for Areas (SEIFA) [27], ‘children’s SES was computed based on their residential postcode. Children’s SES and sex were used only to describe the study population and were not used in analyses of brand awareness given the aim of this study was psychometric testing of a tool rather than quantifying actual brand awareness. The dependent variable was brand awareness, divided into recall and recognition for each of the ‘big 5’ food products as well as total scores. The nomological validity analyses used brand awareness data obtained from Interview 1.

Normality of the data (based on the continuous brand awareness score variable) was confirmed using the Shapiro-Wilk test (*p* = 0.118), therefore parametric tests were used for statistical analyses. A p-value of < 0.05 was considered significant in the final analyses.

### Brand awareness scores

Participants received one point for correctly identifying the brand logo (recall), which constituted precise identification of the brand name and/or branded character, and half a point for correctly matching the product type (recognition). This gave less weighting to the recognition task due to a 25% chance of randomly guessing the correct product with each trial. This scoring system gave a total possible composite multi-brand awareness score of 45 (30 for recall, 15 for recognition) for the full ABAI and 18 (12 for recall, 6 for recognition) for the ABAI-a.

### ‘Nomological’ validity of ABAI

Nomological validity measures a tool’s correlation with variables that are expected to be related, as based on theoretical arguments. Brand awareness is expected to be a reflection of a child’s prior food marketing exposure [12], therefore variables that were predicted to be associated with brand awareness are exposure to forms of food marketing, of which TV advertising, point-of-purchase and packaging (from supermarket attendance), and internet-based marketing were included. Age was the key variable of interest as previous studies have consistently found that age is the most significant determinant of brand awareness, compared with other variables [13, 18, 19, 28].

Pearson’s correlation coefficients were used to examine the relationship between brand awareness and the continuous variables of age, minutes of TV viewing/week (commercial, non-commercial and pay TV, separately), internet usage, total screen-time, number of TV’s in the household and ‘total consumption score’ for the ‘big 5’ products.

One-way ANOVAs were used to explore differences in the mean brand awareness scores between children with varying consumption levels. This was completed for each of the ‘big 5’ categories; e.g. comparing if mean brand awareness scores for fast food brands (recall and recognition, separately and combined) differed between children with varying frequencies of fast food consumption. This test was also used for other ordinal, categorical variables using the same 4-point scale, including frequency of eating dinner while watching TV and supermarket attendance, to determine any between-group differences in mean brand awareness scores. A linear regression model was also used to assess whether a linear relationship existed between the variables of age, commercial TV viewing, consumption score and brand awareness.

### Agreement between the ABAI and ABAI-a

The consistency between the full-ABAI and ABAI-a was assessed by first multiplying scores from the ABAI-a by 2.5 for equivalence to the ABAI. The following statistical tests were used: i) Comparison of means using paired t-tests; ii) Pearson’s Correlation Coefficients to assess the strength of a relationship between the scores for the ABAI-a and ABAI; and iii) Bland-Altman Limits of Agreement (LOA) plots, to assess the mean agreement between the two measurement tools using procedures outlined by Bland and Altman [29].

### Reliability

The test-retest reliability of the full ABAI was assessed by comparing the consistency in responses from children who completed the full ABAI for Interviews 1 and 2. Intra-class Correlation Coefficients (ICC) were used, which analyse the consistency in results obtained between two differing trials using the same measurement tool [30].

### Sensitivity analysis

Paired-samples t-tests were performed to assess any differences in the mean responses obtained from matched parents and child questionnaires for children’s ‘total cumulative TV viewing’ and ‘total internet usage’. One-way ANOVAs were used to assess any differences in children’s mean brand awareness (from child interviews) for each of the ‘big 5’ categories and parent-reported frequency of consumption. A composite ‘frequency of consumption’ score was created by adding together the frequency categories based on parent report and Pearson’s Correlation Coefficients were used to measure the strength of a relationship between these scores and total brand awareness.

Total TV viewing, internet usage, and total screen-time were compared with brand awareness scores using Pearson’s Correlation Coefficients. A one-way ANOVA was used to assess whether there were any differences in brand awareness between children who had differing frequencies of supermarket attendance, based on parent report. Linear regression was performed in order to determine whether a linear relationship existed between the variables of cumulative TV viewing, consumption score and brand awareness.

## Results

### Sample characteristics

Of the 66 children (7–12 years) who returned signed parental consent, a total of sixty participated in at least one component of the study; one child was excluded due to a developmental delay, three refused to participate and two did not return to centres during data collection (91% child response rate). Consequently, our sample contained no twelve year olds. Data from 27 children were used for the reliability study and 30 children were used to assess the agreement between the short and long versions of the tool. Three participants only completed the first interview but were included in the ‘nomological’ validity analyses. Twenty-two parent questionnaires were received (37% parent response rate). However, one was excluded as they had not identified their child’s name on the questionnaire.

The study population consisted of 35 boys (58%) and 25 girls (42%). The average age of participants was 8.4 years (SD = 1.3). Based on the ABS SEIFA scores (25), 52% of children were classified as being residents in average relative SES suburbs, 33% in low relative SES suburbs and 9% in high or very high relative SES suburbs.

### ABAI response distribution

Children performed significantly better in the recognition (brand-product association) task compared to the recall (brand naming) task (mean number of correct responses = 22 vs. 14, respectively, *p* < 0.001).

The mean scores for the recall and recognition tasks and for total brand awareness for each age category are shown in Table 1. Fast food was the most highly recalled (mean = 5.2, SD = 1.2) and recognised (mean = 5.6, SD = 0.7) ‘big 5’ product category (Table 2).

**Table 1:** Mean score of responses for the brand recall, brand recognition and total brand awareness for each age category.

Age (Years)	Recall Mean (SD)^♦^	Recognition Mean (SD)^ʈ^	Total Brand Awareness Mean (SD)
7 (*n* = 21)	10.7 (5.4)	9.8 (2.3)	20.3 (7.0)
8 (*n* = 14)	12.6 (4.7)	10.1 (1.6)	22.8 (6.0)
9 (*n* = 11)	14.5 (5.9)	10.9 (3.0)	25.5 (8.8)
10 (*n* = 11)	19.9 (1.5)	12.8 (2.0)	32.7 (6.4)
11 (*n* = 3)	22.7 (2.5)	13.7 (1.0)	36.3 (3.5)
**Total (60)**	**14.2 (6.3)**	**10.8 (2.5)**	**25.0 (8.4)**

^♦^Average scores for the brand naming task (1 point for each correct answer), maximum score 30

^ʈ^ Average scores for the brand-product association task (0.5 point for each correct answer), maximum score 15

**Table 2:** Mean number of correct responses and mean score of responses for the recall and recognition tasks for each of the ‘big 5’ categories.

Big 5 Category	Recall: mean number of correct responses (SD)	Recognition*: mean number of correct responses (SD)
Fast Food	5.2 (1.2)	5.6 (0.7)
Confectionary	3.0 (1.6)	4.8 (1.3)
Sugary Drinks	2.9 (2.0)	4.1 (1.5)
Sugary Cereals	1.7 (1.5)	3.6 (1.5)
Salted Snacks	1.3 (1.6)	3.4 (1.5)

* Each correct response for recognition allocated a score of 0.5

Based on the mean of the scores from children who completed the full ABAI for both Interviews 1 and 2 (n = 27), children obtained significantly higher brand awareness scores in the second trial (mean = 26.5 vs. 29.6, *p* < 0.001).

### ‘Nomological’ validity

As expected, brand awareness scores significantly increased with age (*r* = 0.605, *p* < 0.01) (Table 1). Based on the dietary intake data reported by children, no significant differences in total brand awareness scores were identified between the groupings derived from ‘frequency of consumption’ for any of the ‘big 5’ product categories (Fast Food (F (1, 58) = 0.719, *p* = 0.400), Sugary Cereals (F (3, 56) = 0.919, *p* = 0.437), Salted Snacks (F (3, 56) = 2.356, *p* = 0.082), Confectionary (F (3, 56) = 0.185, *p* = 0.906), Sugary Drinks (F (3, 56) = 2.134, *p* = 0.106). Similarly, no statistically significant differences were found for either recall or recognition, separately, of the ‘big 5’ brands and frequency of consumption for each of the products. The total composite ‘frequency of consumption score’ was not significantly correlated with total brand awareness (*r* = 0.094, *p* = 0.473).

Pearson’s correlational analyses revealed no significant relationships between total recall (*r* = −0.003, *p* = 0.981), total recognition (*r* = -0.218, *p* = 0.094) or total brand awareness score (*r* = −0.47, *p* = 0.724) and cumulative minutes of commercial TV viewing per week. Similarly, no correlation was found between total recall (*r* = 0.018, *p* = 0.888), total recognition (*r* = 0.087, *p* = 0.510) or total brand awareness (*r* = 0.049, *p* = 0.708) and cumulative minutes of pay TV viewing per week. As expected there was no significant relationship between cumulative non-commercial TV viewing and total brand awareness (*r* = −0.117, *p* = 0.374). Brand awareness was not significantly correlated with the number of TVs in a child’s household (*r* = 0.009, *p* = 0.948), or cumulative minutes of internet usage per week (*r* = −0.085, *p* = 0.516). Even when total commercial TV (including pay TV) and internet usage were combined to represent total screen-time exposure, no correlation was found between this and total recall (*r* = -0.039, *p* = 0.767), total recognition (*r* = -0.148, *p* = 0.259) or total brand awareness (*r* = -0.055, *p* = 0.675).

There were no differences in brand awareness scores based on frequency of eating dinner while watching TV (F (3, 56) = 0.372), *p* = 0.774) or frequency of supermarket attendance (F (2, 57) = 0.512, *p* = 0.599) (Table 3). TV and food/drink consumption were not associated with the outcome of awareness in univariate linear regression models. Age was associated with the outcome of awareness (F1,58 = 30.70, *p* < 0.001). Each year of age was associated with a 3.9 unit increase in awareness score. Age accounted for 35% of the variation in awareness score across the group.

**Table 3:** Linear regression outputs.

Variable	Unstandardized β	t-value	95% CI	R^2^	P value
Age	3.884	5.54	2.481, 5.288	0.346	<0.001
TV viewing ^†^	-0.002	-0.821	-0.008, 0.004	0.011	0.415
Internet usage	-0.002	-0.703	-0.009, 0.004	0.008	0.485
Total screen time	-0.001	-0.560	-0.006, -0.003	0.005	0.578
Food consumption score	0.407	0.971	-0.432, 1.247	0.016	0.335

^†^ Cumulative minutes per week

### Sensitivity analysis

Paired-samples t-tests revealed that there were statistically significant differences is the mean responses between the 21 parent and child questionnaires for ‘total TV viewing’ (*p* = 0.005) and ‘total internet usage’ (*p* = 0.028). Based on responses from the parent questionnaires, there were no statistically significant differences in child awareness of the ‘big 5’ products and parent-reported frequency of consumption for each of the ‘big 5’ product categories (Fast Food (F (1, 19) = 0.128, *p* = 0.725), Sugary Cereals (F (2, 18) = 1.783, *p* = 0.197), Salted Snacks (F (3, 17) = 0.173, *p* = 0.913), Confectionary (F (3, 17) = 0.891, *p* = 0.466), Sugary Drinks (F (2, 18) = 1.838, *p* = 0.188). Similarly, there were no statistically significant difference in either recall or recognition of the ‘big 5’ brands and parent-reported frequency of consumption for each of the products. The total composite ‘frequency of consumption’ score, computed from parent data, was not significantly associated with children’s total brand awareness scores (PCC = −0.056, *p* = 0.815).

There were no statistically significant correlations between children’s total brand awareness and parent-reported TV viewing (PCC = 0.056, *p* = 0.813), internet usage (PCC = 0.257, *p* = 0.274) or total screen-time for children (PCC = 0.199, *p* = 0.400). Nor were there any statistically significant differences in child brand awareness based on frequency of supermarket attendance (F (1, 18) = 0.463, *p* = 0.505), as reported by parents. Total screen time, and separately TV and Internet, were not associated with awareness outcome (all P > 0.05) in univariate linear regression models (Table 3).

### Relative validity

**Fig 2:**
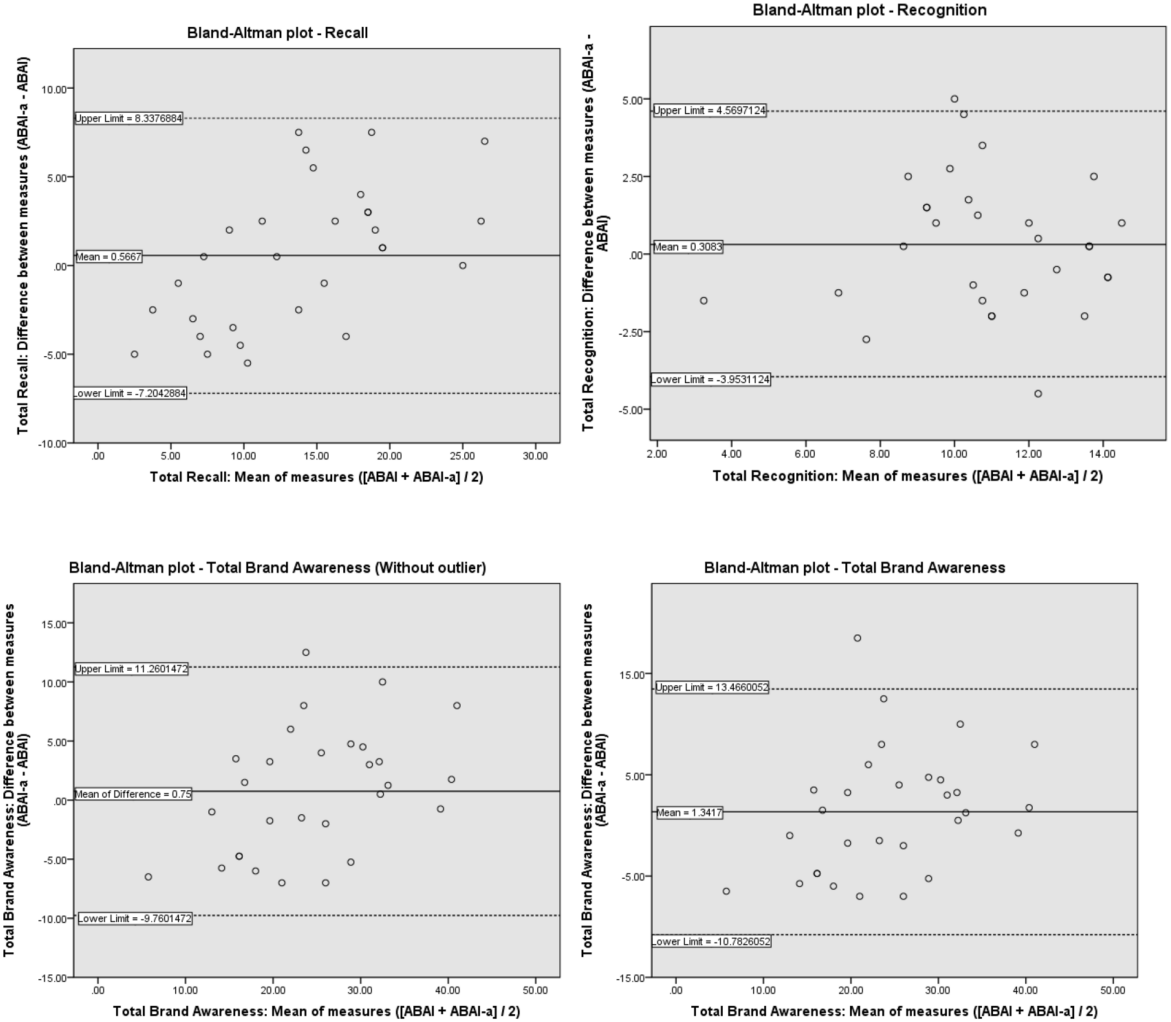
Bland-Altman plots assessing the validity of the ABAI-a v. the ABAI for recall, recognition and total brand awareness (with and without one outlier) among children (n 30) aged 7–11 yrs, Illawarra, Australia, July-Aug 2014. Plots show the mean difference (centre horizontal lines), and the 95% limits of agreement (dotted lines) for: (a) brand recall, (b) brand recognition, (c) total brand awareness (with outlier), (d) total brand awareness (outlier removed).

Bland-Altman plots were constructed to show the mean agreement between the two versions of the tool (full and abbreviated) for total recall, total recognition, total brand awareness including one outlier, and total brand awareness excluding one outlier (Fig 2). The outlier was excluded based on the considerable difference (18.5 points) between the ABAI score obtained in Interview 1 and the equivalent ABAI-a score from Interview 2.

All plots display a positive mean difference between the ABAI-a and ABAI, indicating that the ABAI-a tended to give a higher estimate of brand awareness. Table 4 presents results of the agreement between the ABAI and ABAI-a. Overall, the two methods showed good agreement, as the mean of the differences for each plot were close to zero, indicating that the two ABAI versions were not systematically producing largely differing results. Additionally the percentage difference between the two methods was less than 4% of the ABAI mean for all measures, and at the group level, the ABAI-a had the tendency to overestimate total brand awareness by a mere 3% (when one considerable outlier was removed).

**Table 4:** Consistency between responses for the ABAI and ABAI-a.

Measure	Difference ABAI-a—ABAI Mean (SD)	95% LOA^ŧ^	% difference^ʈ^
**Recall**	0.57 (3.96)	-7.20–8.34	4.17%
**Recognition**	0.38 (2.174)	-3.95–4.57	2.90%
**Total Brand Awareness**	1.34 (6.19)	-10.78–3.47	5.62%
**Total Brand Awareness (without outlier)**	0.75 (5.36)	-9.76–11.26	3.09%

LOA—Limits of agreement; ABAI—Australian Food Brand Awareness

^ŧ^Mean difference ± 1.96 × SD of difference

^ʈ^(ABAI-a Mean—ABAI Mean/ABAI Mean)*100

*p* < 0.05*; *p* < 0.01**

The 95% LOA show that at the individual level, the differences between the two methods were not exceptionally large. Overall the ABAI-a could underestimate total brand awareness (with a total possible score of 45) by a maximum of 10 points and overestimate by a maximum of 11 points in 95% of the population. In terms of the recall task (total possible score of 30), the ABAI-a could overestimate recall scores by 8 and underestimate by 7 relative to the full ABAI, in 95% of the population. For recognition (total possible score of 15), the ABAI-a could overestimate recognition by 5 and underestimate by 4 relative to the full ABAI, in 95% of the population. In the case of the recall task (*p* = 0.001) and total brand awareness scores (*p* < 0.05), there was a slight tendency for the differences between the two methods to increase as mean brand awareness score increased.

Paired t-tests revealed that there was no statistically significant difference in the mean scores obtained from the two versions of the tool for the recall task (*p* = 0.440), recognition task (*p* = 0.444), or total brand awareness score (*p* = 0.244). Pearsons Correlation Coefficients showed good to excellent correlation between the two versions of the tool, with regards to the recall task (PCC = 0.874, *p* < 0.01), recognition task (PCC = 0.678, *p* <0.01), and total brand awareness (PCC = 0.784, *p* < 0.01).

### Reliability

Table 5 shows the ICCs for recall, recognition and total brand awareness between the two administrations of the full ABAI. The ICCs revealed excellent test-retest reliability for detecting overall brand awareness, with a significant r value of 0.927 (*p* < 0.01).

**Table 5:** Test-retest reliability of the ABAI (n = 27).

Measure	First ABAI Mean (SD)	Second ABAI Mean (SD)	ICC (*p* value)
**Total Recall**	15.4 (7.03)	17.6 (6.90)	0.941 (*p* < 0.01)
**Total Recognition**	11.1 (2.35)	12.0 (2.20)	0.831 (*p* < 0.01)
**Total Brand Awareness**	26.5 (9.16)	29.6 (8.57)	0.927 (*p* < 0.01)

## Discussion

This study aimed to develop a tool for measuring food/drink brand awareness in Australian children, aged 7–12 years, as a proxy for their prior food marketing exposure, based on the existing International Brand Awareness Instrument [18–21]. The new ABAI was found to detect varying levels of brand awareness in this study population, both across age groups and also for food product categories. Consistent with previous IBAI studies [18–21], the ABAI facilitated more correct responses for the recognition (product-association) task compared with the recall (brand naming) task.

Children were able to recall fast food brands at a higher frequency compared with other product categories, with 97% of participants being able to effectively identify brand names when presented with a modified Subway and McDonald’s logo. This parallels results of previous research using similar logo recognition tasks [12, 31], which also found high rates of recognition for fast food brands compared with other types of food/non-food related brands. This demonstrates the particular effectiveness of fast food marketing campaigns to engage children’s attention and integrate brand information into memory. Content analyses of the extent and nature of TV food advertising have found that unhealthy food ads are dominated by fast food restaurants, which may explain children’s high awareness of these products [32].

To address limitations in previous literature using brand awareness tools, the current study explored the validity and reliability of these tools. ‘Nomological’ validity analyses revealed conflicting findings. Consistent with previous research [12, 13, 18, 19, 33, 34], brand awareness significantly increased with age, suggesting the effects of longer exposure to food marketing as well as increased information processing abilities. This served as the main predictor of brand awareness.

As brand awareness is supposedly a reflection of food marketing exposure, it was expected that certain food-marketing related variables, including consumption of frequently promoted foods and TV viewing, would be related to brand awareness. No such associations were found. However, previous studies have similarly found no differences related to frequency of consumption of regularly promoted food products and brand awareness [28, 34]. Although children possess considerable influence over family purchases, parents remain the primary gatekeepers to a child’s food intake [13]. Young children may not have the means to actually purchase food products themselves, and as such, studies investigating the persuasive effects of marketing on children often measure food product requests as opposed to actual eating behaviours [13]. This may explain the lack of correlation between children’s food brand awareness and their actual intake of these items.

Earlier correlational studies that have explored commercial TV exposure in relation to brand recognition have reported weak positive correlations (*r* = 0.16, *p* < 0.05) [13] and differences in brand recognition with varying levels of TV exposures [12, 35]. Conversely, recall has not been shown to be significantly correlated with TV exposure in 4–12 year olds within the literature, with amount of TV viewing only predicting brand recognition [13]. Our study found no relationship for any measure of brand awareness and TV viewing. While the sensitivity analyses using parent-derived data revealed a change in the direction of a relationship between these variables (from negative to a weak positive), these values were not significant, possibly due to a small parent sample (post-hoc power analyses revealed that a sample size of 76 was required to detect a significant relationship between these variables). This sample size was determined by dividing children into two groups of high/low commercial TV viewers based on parent report, using median split. The mean and standard deviation of brand awareness score for each of these groups was calculated. These values were entered into an online sample size calculator, with a power of 80% and alpha of 0.05.

Internet usage and total screen-time were not significantly correlated with brand awareness. Despite the fact that TV promotions are a dominant form of advertising, their prominence is declining [36] and marketers use a wide-range of other promotional channels [14]. Sensitivity analyses also indicate that there may have been issues with child responses for food marketing-related variables.

The repeatability of the IBAI had not been explored in previous studies. The ICC values for the two administrations of the ABAI indicated moderate to excellent agreement between the two measures. However, the number of correct responses for the second interview was significantly higher than the first. It is probable that children were more conscious of brands they encountered in the week between administrations, facilitating more effective recall of the brand name from memory following the second exposure.

In line with previous studies, an abbreviated tool (ABAI-a) was created to reduce participant burden. Based on the Bland-Altman plots, the LOAs indicate that the differences between ABAI and the ABAI-a were not exceptionally large at the individual level. At the group level, the ABAI-a could estimate brand awareness within ± 6% of the full-version, for each of the measures of recall, recognition and total brand awareness. There was a slight tendency for the ABAI-a to overestimate total brand awareness compared with the full-version, which may be attributed to the same effects found in the reliability analysis (i.e. children were more attuned to branding at the second interview). Despite this, the ABAI-a showed good consistency with the full version of the tool.

There are some limitations of the present study. In terms of validity, the novel design of the ABAI makes it difficult to validate in ways other than assessing its ‘content’ validity or comparing brand awareness scores with variables expected to be associated, as seen with ‘nomological’ validity analyses. There is no ‘gold standard’ for measuring awareness with which to assess criterion validity. The arbitrary selection of brands also raises concern as there is no definitive list of brands heavily marketed to children in the Australian context. It is therefore possible that we may have left out brands that are popular and widely promoted within Australia. However, we addressed these concerns by having the list of brands validated with food marketing experts. The study sample did not aim to be representative of all 7–12 year old Australian children. However, the sample size (of both children and parents) used in the analysis of ‘nomological’ validity may have contributed to the unexpected lack of correlation in the results. In addition, the lack of specificity concerning the amount and nature of advertisements to which respondents had been exposed will have affected findings regarding TV viewing in relation to brand awareness. Although we separated viewing into commercial and non-commercial TV, we did not incorporate the exact time of day the viewing occurred, which would also affect the amount of TV advertising a child was exposed to [37]. Similar to this, the nature of the websites the children viewed was not explored for internet usage. For practical purposes, we had to rely on child self-reporting their media usage and food consumption behaviours, and self-reported child data has been known to be potentially inaccurate [24]. While we did attempt to control for inaccurate reporting by also considering parent report, few parents returned the written questionnaire.

Despite these issues, the tool was found to have excellent ‘content’ validity and good ‘nomological’ validity, based on the pattern of correlation between age and brand awareness scores, as well as excellent test-retest reliability. Additionally, the ABAI-a and ABAI demonstrated good consistency, especially at the group level. At the individual level there was a slight bias between the two methods, which could be explained by the fact that children seemed more attuned to the branding images during the second interview. Therefore, when factoring in this effect, the shorter version of the tool shows good agreement with the full version, and could be substituted in future trials. These tools are appropriate to be used in future descriptive studies to assess population brand awareness for children aged 7–12 years. Such population studies would give an indicator of children’s cumulative and relative awareness of major food brands, and relationship with affective and behavioural outcomes may be explored. Additionally, the tool can be useful in experimental trials that expose groups of children to advertising conditions, as a measure of baseline brand awareness, to explore the impact of episodic exposure to promotions on food-related attitudes and choice. Such baseline awareness is likely an important modifier of responses to episodic marketing exposures. Developing instruments, such as the IBAI and ABAI, specific to each country, is important for studying the changes in our food environment, and elucidating the potential role that unhealthy food marketing has had in the rising prevalence of childhood obesity.

## Supporting Information

S1 Table The Australian Brand Awareness Instrument. ** The 12 brands and associated products included in the flash cards for the ABAI-a.
(DOCX)

## References

[1] Lobstein T, Baur L, Uauy R for the IOTF Childhood Obesity Working Group (2004) Obesity in children and young people: a crisis in public health. Obes Rev 5(1):4–85 15096099 10.1111/j.1467-789X.2004.00133.x

[2] Australian Bureau of Statistics (07 June 2013) Australian Health Survey: updated results, 2011–2012. Available: http://www.abs.gov.au/ausstats/abs@.nsf/Lookup/4364.0.55.003main+features12011-2012. Accessed 15 March 2014.

[3] Swinburn B, Egger G, Raza F (1999) Dissecting obesogenic environments: the development and application of a framework for identifying and prioritizing environmental interventions for obesity. Prev Med 29(6):563–570 10600438 10.1006/pmed.1999.0585

[4] Harris J, Pomeranz J, Lobstein T, Brownell K (2009) A crisis in the marketplace: how food marketing contributes to childhood obesity and what can be done. Annu Rev Public Health, 30(1):211–225 18976142 10.1146/annurev.publhealth.031308.100304

[5] World Health Organization (2010) Set of recommendations on the marketing of foods and non-alcoholic beverages to children. Available: http://whqlibdoc.who.int/publications/2010/9789241500210_eng.pdf. Accessed 15 March 2014

[6] United Nations (2010) Private sector should stop marketing unhealthy foods to children, urges secretary-general in message to meeting on non-communicable diseases. Available: http://www.un.org/News/Press/docs/2014/sgsm16013.doc.htm. Accessed 16 March 2014

[7] World Health Organization (2012) A comprehensive global monitoring framework, including indicators, and a set of voluntary global targets for the prevention and control of non-communicable diseases. Available: http://www.who.int/nmh/events/2012/discussion_paper3.pdf. Accessed 16 March 2014

[8] Aaker D (1991) Managing brand equity: Capitalizing on the value of a brand name. New York: The Free Press 224 p.

[9] Connor S (2006) Food-related advertising on preschool television: Building brand recognition in young viewers. Pediatrics 118:1478–1485 17015538 10.1542/peds.2005-2837

[10] Kelly B, King L, Chapman K, Boyland E, Bauman A, Baur L (2015) A hierarchy of food promotion effects: identifying methodological approaches and knowledge gaps. J Pub Health, 105(4):86. 10.2105/AJPH.2014.302476 PMC4358159 25713968

[11] Story M, French S (2004) Food advertising and marketing directed at children and adolescents in the US. Int J Behav Nutr Phys Act 1(1):3 15171786 10.1186/1479-5868-1-3 PMC416565

[12] Fischer P, Schwartz M (1991) Brand logo recognition by children aged 3 to 6 years. JAMA, 266(22):31–45 1956101

[13] Valkenburg P, Buijzen M (2005) Identifying determinants of young children’s brand awareness: Television, parents and peers. J Appl Dev Psychol, 26(4):456–468

[14] Cairns G, Angus K, Hastings G, Caraher M(2013) Systematic reviews of the evidence of the nature, extent and effects of food marketing to children. A retrospective summary. Appetite, 62:209 10.1016/j.appet.2012.04.017 22561190

[15] Robinson T, Borzekowski D, Matheson D, Kraemer H (2007) Effects of fast food branding on young children’s taste preferences. Arch Pediatr Adolesc Med, 161(8):792–797 17679662

[16] Roberts M, Pettigrew S (2007) A thematic content analysis of children’s food advertising. IJA, 26(3):357

[17] Boyland E, Harrold J, Kirkham T, Halford J(2012) Persuasive techniques used in television advertisements to market foods to UK children. Appetite, 58(2): 658–664 10.1016/j.appet.2011.11.017 22133361

[18] Forman J, Halford J, Summe H, MacDougall M, Keller K e (2009) Food branding influences *ad libitum* intake differently in children depending on weight status. Results of a pilot study. *Appetite*, 53(1):76–83 19481125 10.1016/j.appet.2009.05.015

[19] Franchin L, Zobec F, Ghidina M, Stefanini G, Berchialla P, Gregori D (2012) Measuring brand awareness as a component of eating habits in children: the development of the IBAI (International Brand Awareness Instrument) Italian version. *Progress in Nutrition* 2012, 14(2):132–140

[20] Vecchio M, Berchialla P, Didier N, Cayul O, Valdenegro D, Ghidina M, et al (2013) The IBAI instrument to assess brand awareness toward food in children: the Chile adaptation. Open Obes J, 5(1):30–35

[21] Vecchio M, Ghidina M, Gulati A, Berchialla P, Paramesh E, Gregori D (2014) Measuring brand awareness as a component of eating habits in Indian children: The development of the IBAI questionnaire. Indian J Pediatr, 81(1):23 24916131 10.1007/s12098-014-1447-y

[22] Huang R, Sarigöllü E (2012). How brand awareness relates to market outcome, brand equity, and the marketing mix. J Bus Res;65(1):92–9

[23] NSW Ministry of Health (2010) NSW Schools Physical Activity and Nutrition Survey (SPANS) 2010: Full report. Available: http://tinyurl.com/pj8pypj. Accessed 17 March 2014.

[24] Fuchs M (2008) The reliability of children’s survey responses: the impact of cognitive functioning on respondent behaviour. Symposium, 11:522–530

[25] Bahn K (1986) How and when do brand perceptions and preferences first form? A cognitive developmental investigate. J Consum Res, 13(3):382–393

[26] Carter O, Patterson L, Donovan R, Ewing M, Roberts C (2011) Children’s understanding of the selling versus persuasive intent of junk food advertising: implications for regulation. Soc Sci Med, 72(6):962–968 10.1016/j.socscimed.2011.01.018 21349621

[27] Australian Bureau of Statistics (2011) Socio-economic Indexes for Areas 2011. Available: http://www.abs.gov.au/ausstats/abs@.nsf/mf/2033.0.55.001/. Accessed 20 August 2014.

[28] Ueda P, Tong I, Viedma C, Chandy S, Marrone G, Simon A et al (2012) Food marketing towards children: brand logo recognition, food-related behaviour and BMI among 3–13 year olds in a South Indian town. PLoS ONE, 7(10):1–7 10.1371/journal.pone.0047000 PMC3474829 23082137

[29] Bland M, Altman D (2010) Statistical methods for assessing agreement between two methods of clinical measurement. Int J Nurs Stud, 47(8):931–936

[30] Karras D (1997) Statistical methodology: II. Reliability and validity assessment in study design, Part A. Acad Emerg Med, 4(1):64–71 9110015 10.1111/j.1553-2712.1997.tb03646.x

[31] Arredondo E, Castaneda D, Elder J, Slymen D, Dozier D (2009) Brand name logo recognition of fast food and healthy food among children. J Commun Health, 34(1):73–78 10.1007/s10900-008-9119-3 18830690

[32] Kelly B, Smith B, King L, Flood V, Bauman (2007) Television food advertising to children: the extent and nature of exposure. Public Health Nutr, 10(11):1234–1240 17381920 10.1017/S1368980007687126

[33] Mizerski R (1995) The relationship between cartoon trade character recognition and attitude toward product category in young children. The Journal of Marketing 1995, 59(4):58–70

[34] Kopelman C, Roberts L, Adab P (2007) Advertising of food to children: is brand logo recognition related to their food knowledge, eating behaviours and food preferences? J Public Health, 29(4):358–367 10.1093/pubmed/fdm067 17998259

[35] Goldberg M (1990) A quasi-experimental assessing the effectiveness of TV advertising directed to children. J Marketing Res, 27(4):445–454

[36] King L, Hebden L, Grunseit A, Kelly B, Chapman K (2013) Building the case for independent monitoring of food advertising on Australian television. Public Health Nutr, 16(12):2249 10.1017/S1368980012004429 23034219 PMC10271793

[37] Kelly B, Chapman K, King L & Hebden L (2011) Trends in food advertising to children on free-to-air television in Australia. Aust N Z J Public Health 2011, 35(2): 131–134 10.1111/j.1753-6405.2011.00612.x 21463408

