# Supplementary material for: Measuring Food Brand Awareness in Australian Children: Development and Validation of a New Instrument
Source: PLoS One. 2015 Jul 29;10(7):e0133972. doi: 10.1371/journal.pone.0133972 (PMC4519263; doi:10.1371/journal.pone.0133972)
Supplement: S1 Table — ** The 12 brands and associated products included in the flash cards for the ABAI-a. (DOCX) [file pone.0133972.s001.docx]

Table S1: The Australian Brand Awareness Instrument

| No | Brand | Answer | Product 1 (A) | Product 2  (B) | Product 3  (C) | Product 4  (D) |
| --- | --- | --- | --- | --- | --- | --- |
| 1 | Cadbury** | B | Nutrigrain | Chocolate | Pringles | Coke |
| 2 | Cheezels** | C | Frosties | Smiths chips | Cheezels | M & Ms |
| 3 | Coca Cola | A | Coke | Coco Pops | Milo drink | KFC chicken |
| 4 | Coco pops (Kelloggs) | A | Coco Pops | Chocolate | Coke | Nutella on Toast |
| 5 | Corn Flakes  (Kelloggs) | D | Chicken & chips | Dorritoes | Kinder egg | Corn Flakes |
| 6 | Dominoes | C | Chicken & chips | Big Mac | Pizza | Subway sandwich |
| 7 | Dorrito’s** | B | Corn Flakes | Dorrito’s | Red Bull | Oreos |
| 8 | Fanta** | A | Fanta | Milo & milk | Corn Flakes | Twisties |
| 9 | Froot Loops** | D | Cheezels | Fanta | Kinder egg | Froot loops |
| 10 | Frosted Flakes  (Kelloggs) | A | Frosties | Twisties | Mars Bar | Energy drink |
| 11 | Hungry Jacks** | C | Smiths chips | Kinder egg | Whopper burger | Froot Loops |
| 12 | Kettle Chips | D | Cheezels | Chicken & Chips | Energy drink | Kettle Chips |
| 13 | Kinder** | B | Nutrigrain | Kinder egg | KFC chicken | Kettle Chips |
| 14 | KFC** | A | KFC chicken | Pizza | Pringles | Oreos |
| 15 | M & Ms | D | Coco Pops | Smiths chips | Kinder egg | M & Ms |
| 16 | Mars | C | Dorritoes | Pepsi | Mars Bar | Nutrigrain |
| 17 | McDonalds | D | KFC chicken | Pizza | Pringles | Big Mac |
| 18 | Monster | A | Energy drink | Nutrigrain | Big Mac | Mars bar |
| 19 | Milo | C | Pepsi | Dorritoes | Milo drink | Oreos |
| 20 | Nutella | C | Coke | Dorritoes | Nutella on Toast | Coco Pops |
| 21 | Nutrigrain**  (Kelloggs) | C | Subway Sandwich | Pepsi | Nutrigrain | Kettle chips |
| 22 | Oreos | B | Coco Pops | Oreos | Pepsi | Cheezels |
| 23 | Pepsi** | B | Nutrigrain | Pepsi | KFC chicken | Mars bar |
| 24 | Pringles** | A | Pringles | KFC chicken | Twisties | Oreos |
| 25 | Red Bull** | D | Whopper burger | Milo drink | Rice Bubbles | Energy drink |
| 26 | Red Rooster | C | Kinder egg | Pizza | Chicken & chips | Corn Flakes |
| 27 | Rice Bubbles | A | Rice Bubbles | Milo drink | Smiths Chips | Oreos |
| 28 | Smiths Chips | C | Cheezels | Fanta | Smiths Chips | Mars Bar |
| 29 | Subway | B | Pringles | Subway Sandwich | Pizza | KFC Chicken |
| 30 | Twisties | D | Pringles | KFC chicken | Energy drink | Twisties |

** The 12 brands and associated products included in the flash cards for the ABAI-a
